# Tumor characteristics and prognosis in familial breast cancer

**DOI:** 10.1186/s12885-016-2962-1

**Published:** 2016-11-29

**Authors:** G. Arpino, M. Pensabene, C. Condello, R. Ruocco, I. Cerillo, R. Lauria, V. Forestieri, M. Giuliano, C. De Angelis, M. Montella, A. Crispo, S. De Placido

**Affiliations:** 1 Department of Clinical Medicine and Surgery, University of Naples Federico II, Naples, Italy; 2 The Lester and Sue Smith Breast Center, Baylor College of Medicine, Houston, Texas USA; 3 Department of Epidemiology, Istituto Nazionale Tumori Pascale, Naples, Italy

## Abstract

**Background:**

Approximately 5–10% of breast cancers are hereditary and their biology and prognosis appear to differ from those of sporadic breast cancers. In this study we compared the biological features and clinical characteristics of non metastatic breast cancer in patients with BRCA mutations versus patients with a family history suggesting hereditary breast cancer but without BRCA mutations (BRCA wild type) versus patients with sporadic disease, and correlated these findings with clinical outcome.

**Methods:**

We retrieved the clinical and biological data of 33 BRCA-positive, 66 BRCA-wild type and 1826 sporadic breast cancer patients contained in a single institution clinical database between 1980 and 2012. Specifically, we recorded age, tumor size, nodal status, treatment type, pattern of relapse, second primary incidence, outcome (disease-free survival and overall survival), and biological features (estrogen receptor [ER], progesterone receptor [PgR], tumor grade, proliferation and c-erbB2 status). Median follow-up was 70 months.

**Results:**

BRCA-positive patients were significantly younger than sporadic breast cancer patients, and less likely to be ER-, PgR- or c-erbB2-positive than women with BRCA-wild type or sporadic breast cancer. Tumor size and grade, nodal status and proliferation did not differ among the three groups. Rates of radical mastectomy were 58, 42 and 37%, and those of conservative surgery were 42, 58 and 63% in women with BRCA-positive, BRCA-wild type and sporadic breast cancer (*p* = 0.03), respectively. The incidence of contralateral breast cancer was 12, 14 and 0% (*p* <0.0001) and the incidence of second primary tumors (non breast) was 9, 1 and 2% (*p* <0.0001) in BRCA-positive, BRCA-wild type and sporadic breast cancer, respectively. Median disease-free survival in years was 29 in BRCA-wild type, 19 in BRCA-positive and 14 in sporadic breast cancer patients (log-rank = 0.007). Median overall survival in years was not reached for BRCA-wild type, 19 for BRCA-positive and 13 for sporadic breast cancer patients (log-rank <0.0001). At multivariate analyses only BRCA-wild type status was related to a significant improvement in overall survival versus the sporadic breast cancer group (HR = 0,51; 95% CI (0,28–0,93) *p* = 0.028).

**Conclusions:**

The biology and outcome of breast cancer differ between patients with BRCA mutations, patients with a family history but no BRCA mutations and patients with sporadic breast cancer.

## Background

Carcinoma of the breast is a biologically heterogeneous disease. Approximately 5–10% of all breast cancers have a hereditary background. BRCA1 and BRCA2 mutations account for 25–28% of hereditary breast cancers [1]. Young women are more likely to have BRCA-associated breast cancer than to be affected by sporadic disease, and BRCA-positive cancers are usually high grade and lack hormone receptors [2]. Compared with sporadic cancer, the prognosis of BRCA-positive cancer has been reported to be worse [3, 4] not different [5, 6] or better [7]. In about 70% of families with aggregation of breast and ovarian cancers and a pedigree strongly suggesting hereditary breast cancer, the BRCA test was negative and no pathogenic mutation was identified [8–12]. This subgroup of familial non-BRCA mutated breast cancers are very heterogeneous and not well defined in terms of histopathological features, clinical presentation and prognosis. Moreover, little is known about the biological features of BRCA-mutated and of BRCA-wild type breast cancer in relation to their clinical outcome. Therefore, we compared the features of BRCA-mutated, BRCA-wild type and sporadic breast cancer recorded in a large database in the attempt to shed light on their biological phenotype and clinical behavior, and to obtain information that might aid clinical decision making and studies exploring the biological nature of this disease.

## Methods

### Patients’ characteristics

From 1980 to 2012, patients with breast cancer presenting at our Department were screened for family history of cancer and referred to the genetic counseling service, if eligible. To be eligible for cancer genetic counseling, patients had to fulfill one of two criteria: 1) a personal history suggesting a genetic risk (i.e., early onset breast cancer, breast and ovarian cancer in the same subject, and multiple cancers besides breast and ovarian cancers in the same subject); and 2) a significant family history of breast and/or ovarian cancer defined as: (a) at least 2 cases of breast cancer in family members below the age of 50 years or ovarian, peritoneal or tubal cancer in family members at any age; (b) 3 cases of breast cancer or ovarian, peritoneal or tubal cancer in family members at any age; or (c) 1 case of breast cancer in a family member below the age of 50 years or bilateral breast cancer in a family member at any age. Cancer genetic counseling was conducted according to the model previously described [13].

Risk assessment was performed with two predictive models: the clinical criteria of Modena [14] and BRCApro [15, 16]. Patients considered at high risk for a BRCA mutation according to the clinical criteria of Modena and/or a BRCApro test (total score ≥10%) underwent genetic testing for BRCA1 and BRCA2 mutations [17, 18]. Mutation analysis was carried out at the Genetic Oncology Section, Division of Surgical, Molecular and Ultrastructural Pathology, University of Pisa. Genomic DNA was extracted from peripheral blood lymphocytes according to a standard protocol. Mutational screening of BRCA1 and BRCA2 was carried out by direct sequencing. DNA sequencing was carried out directly on PCR-purified products using the BigDye terminator v 3.1 sequencing kit (Applied Biosystems, Foster City, CA) and different primers (primer sequences available upon request). Capillary gel electrophoresis and data collection were carried out with an automated DNA sequencer (ABI PRISM 3100, Applera, Norwalk, CT). Sequence analyses were conducted with the Seq-Scape Software (Applied Biosystems). Mutation nomenclature for the BRCA1 and BRCA2 genes is as used in the BIC database according to the recommendations of GenBank [19].

Data were collected from 1946 women with stage I, IIA, IIB, IIIA or IIIB breast cancer at our Department between 1980 and 2012. Biological and clinical information for this analysis were extracted from a clinical database containing the patients’ charts. No patient enrolled in this study had received bilateral prophylactic mastectomy or prophylactic oophorectomy. A total of 120 patients were eligible for genetic testing for BRCA1 and BRCA2 mutations, according to the criteria described above. The remaining 1826 patients were considered to have a sporadic breast cancer as no clear hereditary pattern was identified. These patients did not undergo genetic testing for BRCA1 and BRCA2 mutations.

Among the 120 patients with a known BRCA status, 33 women were positive for a BRCA1 and/or BRCA2 mutation (BRCA-positive), 66 women were negative for a BRCA1 and/or a BRCA2 mutation (BRCA-wild type) and were considered to have familial non-BRCA mutated breast cancer, and 21 patients had genetic alterations classified “variants of unknown significance”. These patients were excluded from the present study because of the uncertain biologic or clinical relevance of these variants.

The clinical and pathological features of patients with BRCA-positive breast cancer were assessed and compared to those of patients with BRCA-wild type or sporadic breast cancer. Type of treatment, rates of relapse (local and distant), second primary and contralateral breast cancer onset, disease-free survival and overall survival were also compared among the three groups. Institutional Review Board approval was waived as all patients signed informed consent to anonymous data treatment for scientific purpose according to Italian law (art.13, D.lgs. 196/2003).

### Statistical analysis

The patients’ characteristics were compared using the χ^2^ test and an asymptotic Fisher’s exact test for categorical variables and *t* tests for continuous variables. Disease-free survival was calculated as the time from diagnosis to first recurrence (local or distant); cause-specific survival was calculated as the time from diagnosis to death for that cancer, or to censoring if the patient was alive at the last follow-up. Rates of recurrence and second primaries of the three groups (BRCA-positive, BRCA-wild type, sporadic breast cancer) were calculated by dividing the number of events by the total person-time at risk. Unadjusted relative risks (rate ratios) were obtained by dividing the event rate in the screened group by the event rate in the symptomatic groups [20]. The univariate effect of key tumor characteristics on risk of recurrence and death was examined within each patient group. Overall and disease-free survival curves were constructed with Kaplan-Meier analyses and compared using the two-sided log-rank test. The SPSS statistical package version 20.0 (SPSS Inc, Chicago, IL) was used for statistical analysis. A *p*-value <0.05 was considered statistically significant. All patients enrolled in this study had been treated and followed up at our Department.

## Results

### Clinical and pathological characteristics

The data of 1946 patients with non metastatic breast cancer were recorded in the database of the Breast Oncology Division of the University of Naples Federico II between 1980 and 2012. Of these, 1826 patients had sporadic breast cancer, 33 had BRCA-positive cancer (19 BRCA1-mutated and 14 BRCA2-mutated) and 66 had BRCA-wild type breast cancer. As shown in Table 1, BRCA-positive patients were significantly younger than sporadic breast cancer patients, and were less likely to be positive for estrogen receptor (ER), progesterone receptor (PgR) or human epidermal growth factor receptor (c-erbB2) than women with BRCA-wild type or sporadic breast cancer. In detail, in patients with BRCA-positive, BRCA-wild type and sporadic cancer, respectively, 54, 67 and 80% (*p* < 0.0001) of tumors were ER-positive, 54, 68 and 74% (*p* = 0.04) were PgR-positive, and 9, 36 and 17% (*p* = 0.01) were c-erbB2-positive. No statistically significant difference in tumor size, nodal status, grading or proliferation, measured as Ki67, was observed among the three study groups. The type of local and systemic treatment of the primary tumor differed significantly among the three groups (Table 2). Radical mastectomy was more frequent among BRCA-positive and BRCA-wild type patients (58% vs. 42% vs. 37% in BRCA-positive vs. BRCA-wild type vs. sporadic breast cancer, *p* < 0.03). As expected from the distribution of ER, the rate of only chemotherapy as systemic treatment was higher in BRCA-positive patients than in either BRCA-wild type or sporadic breast cancer patients (41% vs. 27% vs. 22%). Rates of adjuvant hormone therapy without chemotherapy were lower in the BRCA-positive patients (13% vs. 23% vs. 24% in BRCA-positive vs. BRCA-wild type vs. sporadic breast cancer patients), and fewer BRCA-wild type patients received chemotherapy followed by endocrine therapy (37% vs. 26% vs. 49% in BRCA-positive vs. BRCA-wild type vs. sporadic breast cancer patients) compared to the other two study groups. All the differences in the type of adjuvant systemic treatment delivered in the three study groups were statistically significant (*p* < 0.0001).

**Table 1:** Patient Demographics and Clinical Characteristics

	BRCA-positive		BRCA-wild type		Sporadic		*p-value*
Number of patients^a^	33		66		1826		
	N	*%*	N	*%*	N	*%*	
*Age at diagnosis*							*<0.0001*
≤ 35	14	*43*	11	*16*	102	*6*	
36–45	10	*30*	33	*50*	421	*23*	
46–55	5	*15*	11	*17*	547	*30*	
≥ 56	4	*12*	11	*17*	756	*41*	
*Tumor size*	*N = 24*		*N = 56*		*N = 1560*		*0.79*
< =2 cm	10	*42*	31	*55*	790	*51*	
> 2 cm, <=5 cm	12	*50*	20	*36*	652	*42*	
> 5	2	*8*	5	*9*	118	*7*	
*Nodal status*	*N = 28*		*N = 59*		*N = 1593*		*0.46*
Node negative	13	*46*	34	*58*	793	*50*	
Node positive	15	*54*	25	*42*	800	*50*	
*Tumor grade*	*N = 20*		*N = 48*		*N = 1435*		*0.07*
*1*	*1*	*5*	*6*	*13*	*82*	*6*	
2	4	*20*	14	*29*	574	*40*	
3	15	*75*	28	*58*	779	*54*	
*ER status*	*N = 26*		*N = 55*		*N = 1545*		*<0.0001*
Positive	14	*54*	37	*67*	1238	*80*	
Negative	12	*46*	18	*33*	307	*20*	
*PgR status*	*N = 26*		*N = 54*		*N = 1531*		*0.04*
Positive	14	*54*	37	*68*	1136	*74*	
Negative	12	*46*	17	*32*	395	*26*	
*Ki67 status*	*N = 21*		*N = 29*		*N = 1079*		*0.87*
Low	6	*29*	10	*35*	327	*30*	
High	15	*71*	19	*65*	752	*70*	
*cerb-B2 status*	*N = 22*		*N = 33*		*N = 1069*		*0.01*
Negative	20	*91*	21	*64*	884	*83*	
Positive	2	*9*	12	*36*	185	*17*	

*ER* estrogen receptor, *PgR* progesterone receptor

^a^The 21 patients carrying variants of unknown significance were excluded from the analysis. Comparisons were made by Chi-square test/asymptomatic Fisher’s exact test

**Table 2:** Type of treatments according to BRCA status

	BRCA-positive		BRCA-wild type		Sporadic		*p*-value
	*N*	%	*N*	%	*N*	%	
*Type of surgery*	*N = 33*		*N = 64*		*N = 1825*		*0.03*
Mastectomy	19	*58*	27	*42*	670	*37*	
Conservative	14	*42*	37	*58*	1155	*63*	
*Adjuvant therapy*	*N = 32*		*N = 66*		*N = 1826*		*<0.0001*
No therapy	3	9	16	24	88	5	
Only CT	13	41	18	27	400	22	
Only HT	4	13	15	23	437	24	
CT + HT	12	37	17	26	901	49	

The 21 patients carrying variants of unknown significance were excluded from the analysis

*CT* chemotherapy, *HT* hormone therapy

### Clinical outcomes

The median follow-up time was 70 months (range: 13–421 months). The development of recurrence differed significantly among the three groups (27% vs. 33% vs. 29% in BRCA-positive vs. BRCA-wild type vs. sporadic breast cancer, respectively). In detail, BRCA-positive-breast cancer patients and BRCA-wild type breast cancer patients were more prone to develop contralateral breast cancer (12% vs. 14% vs. 0% in BRCA-positive vs. BRCA-wild type vs. sporadic breast cancer, respectively) and BRCA-positive breast cancer patients were more prone to develop second non-breast primary tumors (9% vs. 1% vs. 2% in BRCA-positive vs. BRCA-wild type vs. sporadicbreast cancer, respectively). All these data were statistically significant (*p* < 0.0001) (Table 3).

**Table 3:** Recurrence, contralateral breast cancer and second primary rates according to BRCA status

	BRCA-positive		BRCA-wild type		Sporadic		*p*-value
	*N*	%	*N*	%	*N*	%	
	*N = 33*		*N = 66*		*N = 1826*		<0.0001
No events	17	52	34	51	1264	69	
Recurrence	9	27	22	33	522	29	
Contralateral Breast	4	12	9	14	6	0	
Second primary^a^	3	9	1	1	34	2	

The 21 patients carrying variants of unknown significance were excluded from the analysis

^a^Second primaries are defined as all non breast second primaries

**Fig. 1:**
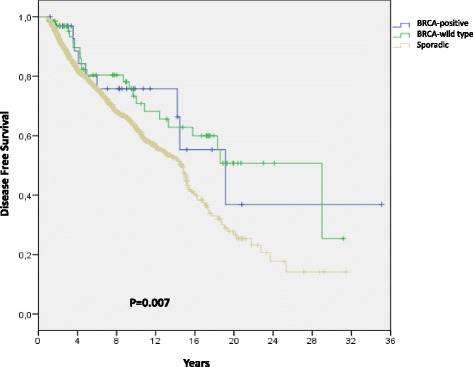
Disease-free survival. Disease-free survival in patients with BRCA-positive, BRCA-wild type or sporadic breast cancer

**Fig. 2:**
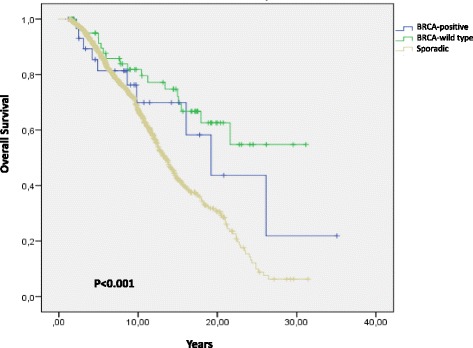
Overall survival. Overall survival in patients with BRCA-positive, BRCA- wild type or sporadic breast cancer

Table 4 shows the recurrence rate and breast cancer mortality in relation to BRCA status. The recurrence rate was 35 per 1000 person-years (95% CI 16–66), 31 per 1000 person-years (95% CI 20–47) and 48 per 1000 person-years (95% CI 45–53), and the rates of overall survival were 32 per 1000 person-years (95% CI 15–59), 22 per 1000 person-years (95% CI 13–35) and 40 per 1000 person-years (95% CI 37–43) in BRCA-positive vs BRCA-wild type vs sporadic breast cancer. As shown in Fig. 1, disease-free survival differed significantly among the three groups (log-rank = 0.007). The median disease-free survival in years was 29 (16–42) for BRCA-wild type, 19 (10–28) for BRCA-positive and 14 (13–15) for sporadic breast cancer patients. Overall survival differed significantly among the three groups (log-rank <0.0001). Median overall survival in years was not reached for BRCA-wild type, 19 (12–26) for BRCA-positive and 13 (12–14) for sporadic breast cancer patients (Fig. 2). However, at multivariate analyses including all three study groups and adjusted for age, year of diagnosis, tumor stage at diagnosis and ER and PgR status, only BRCA-wild type status was related to a statistically significant improvement in overall compared to the sporadic breast cancer group (HR = 0,51; 95% CI 0,28-0,93; *p*=0.028).

**Table 4:** Recurrence and breast cancer mortality in relation to BRCA status

	Person-years^a^	No. of events	Rate x1000 person-years (95% CI)^b^
*DFS*			
BRCA-positive	257	9	35 (16–66)
BRCA-wild type	702	22	31 (20–47)
Sporadic	12721	522	48 (45–53)
OS			
BRCA-positive	312	10	32 (15–59)
BRCA-wild type	814	18	22 (13–35)
Sporadic	14943	598	40 (37–43)

The 21 patients carrying variants of unknown significance were excluded from the analysis

*CI* confidence interval, *DFS* disease-free survival, *OS* overall survival

^a^Rates were calculated from the reference date (i.e. date of diagnosis)

^b^Confidence interval by Haenszel et al. JNCI 1962

## Discussion

Here we provide evidence that the presentation and outcome of breast cancer differ between patients with a strong family history, with or without a BRCA mutation, and patients with sporadic breast cancer. Consistent with previous reports [21–23], most of our BRCA-mutated patients were below the age of 35 years, whereas most patients with sporadic cancer were above the age of 46 years. The incidence of BRCA-wild type breast cancer was higher among women between the ages of 36 and 55 years. In terms of tumor characteristics, we found no difference in tumor size or nodal status at diagnosis among the three study groups. However, tumor biology differed depending on BRCA mutation status. BRCA-positive breast cancers were more likely to be ER-negative and/or PgR-negative and less likely to display c-erbB2 overexpression than either BRCA-wild type or sporadic tumors. Interestingly, there was also a trend, albeit not significant, for BRCA-positive tumors to be grade 3 and to have a higher proliferation rate than the other two types of tumors. These data are consistent with previous studies showing that only a minority of BRCA1-mutated breast cancers have high ER expression rates or overexpress c-erbB2 [24], and that most BRCA-mutated tumors are high-grade and have a higher proliferation fraction compared with sporadic tumors [25, 26].

BRCA-wild type breast cancers have often been reported to be low grade and have biologic features similar to those of sporadic cancers [27, 28]. However, the data are not consistent. In the present study, we found higher c-erbB2 overexpression and a trend towards a higher incidence of grade 1 cancers among BRCA-wild type patients compared to the other two groups.

In our study, patients with BRCA-positive and BRCA-wild type breast cancer were managed, in general, somewhat differently from patients with sporadic disease. In fact, BRCA-positive patients and, albeit to a lesser extent, BRCA-wild type patients underwent mastectomy more frequently than women with sporadic breast cancer. Although this tendency for mastectomy may be related to the bias of the patient and/or the surgeon rather than to BRCA mutation status, which was unknown at diagnosis, the choice of these procedures is also influenced by the patient’s age and tumor phenotypic characteristics. Regarding systemic therapy, because BRCA-positive and BRCA-wild type breast cancer are more frequently steroid receptor-negative tumors, more BRCA-positive and BRCA wild type patients received adjuvant chemotherapy therapy than did sporadic breast cancer patients.

Despite the importance of family history as a risk factor for breast cancer [29], there is disagreement about its impact on prognosis of breast cancer patients with a strong family history associated or not to a BRCA mutation [30–33]. In our study, disease-free and overall survival did not differ significantly between women with BRCA-positive breast cancer and women with sporadic breast cancer. This is in line with reports that survival is not worse in patients with hereditary breast cancer, particularly if adjuvant chemotherapy is administered [34–39]. BRCA mutations render cancer cells more sensitive to DNA breaking agents such as alkylating agents and platinum salts, which are often used in adjuvant treatment. Most of our BRCA-mutation carriers received adjuvant chemotherapy. The increased susceptibility to and the very high rate of adjuvant chemotherapy among BRCA-mutation carriers in our series may have contributed to their not worse prognosis compared to patients with sporadic breast cancer. Interestingly, at multivariate analyses, overall survival was better in BRCA-wild type breast cancer patients than in patients with sporadic breast cancer. Further studies are needed to clarify the genetic background of these tumors and, eventually, to determine whether their increased genetic instability results in a better outcome of anticancer therapies.

In line with previous studies showing that BRCA-mutated breast cancer patients have a strong lifetime risk of developing a second primary cancer [40–43], we found that the incidence of second primary cancers was significantly higher in BRCA-positive patients than in the other two groups. Not surprisingly, we also found that the most frequent second primary in BRCA carrier patients was breast cancer in the contralateral breast. Estimates of contralateral breast cancer incidence among BRCA mutated breast cancer patients varies from 15 to 40% within 10 years [39, 44–48]. None of our BRCA-positive patients underwent prophylactic bilateral mastectomy and rate of contralateral breast cancer in this subgroup was about 12%.

Little is known about the contralateral breast cancer risk in women with BRCA-wild type breast cancer. However, despite the lack of its efficacy, prophylactic bilateral mastectomy after a first breast cancer is being increasingly requested not only by mutation carriers but also by patients with familial non-BRCA mutated breast cancer, especially if the family history strongly suggests hereditary disease [49–52]. In our series, the incidence of a second primary among BRCA-wild type patients was about 15%. Most second primaries were contralateral breast cancer (14%). This rate is higher than reported previously [42, 53]. Several studies have shown that contralateral breast cancer risk in familial non-BRCA-mutated breast cancer patients varies depending on the patient’s age at the first breast cancer diagnosis [42, 48, 54]. In fact, women older than 50 years when diagnosed with breast cancer had a significantly lower risk of contralateral breast cancer (cumulative incidence of contralateral breast cancer 12.9%) than women diagnosed before the age of 40 years (cumulative incidence of contralateral breast cancer 28.4%) [42, 48, 54]. In our BRCA-wild type patients, the young age at diagnosis (most patients were below the age of 45 years) and the absence of prophylactic bilateral mastectomy may have accounted for the increased incidence of contralateral breast cancer we found in this subgroup.

Our study has several strengths. It is a single institution study of the prognostic effects of BRCA mutations and a family history suggestive of hereditary disease on breast cancer outcome in women monitored for almost 15 years. All patients with a family history suggestive of hereditary disease underwent genetic counseling and risk assessment. Genetic testing was conducted only if the patient was considered at high risk for a BRCA mutation according to the clinical criteria of Modena [14] and BRCA pro [15, 16].

The limits of our study are the retrospective nature of the analyses and the relatively low numbers of BRCA1 and BRCA2 mutation carriers identified. Indeed, the stringent criteria used to decide whether to conduct genetic testing (genetic testing was carried out only if patients were considered at high risk for a BRCA mutation) and the prevalence of Caucasian not Ashkenazi patients may account for the relatively low incidence of BRCA mutation found in our dataset. Importantly, the low number of events that occurred in these carriers may have affected the precision of our analyses. Finally, we did not examine the effects of breast surgery (lumpectomy *versus* mastectomy) or of different types of adjuvant chemotherapies on locoregional events or other outcomes.

## Conclusions

Breast cancers in BRCA-mutation carriers and BRCA-wild type patients differ from those in women with sporadic breast cancers in terms of age at presentation, hormone receptor and c-erbB2 status, type of local and systemic adjuvant therapy received and second primary incidence. These clinical and biological differences translated into outcome differences with a better overall survival in the BRCA-wild type patients than in patients with sporadic breast cancer. Notably, there was a high incidence of contralateral breast cancer in patients with BRCA-wild type breast cancer. Should these results be confirmed by other studies, prophylactic bilateral mastectomy may become a reasonable option in this subset of patients.

## Abbreviations

CIs: Confidence intervals
DFS: Disease free survival
ER: Estrogen receptor
HR: Hazard ratio
OS: Overall survival
PgR: Progesteron receptor
